# Cardiovascular phenotype in Smad3 deficient mice with renovascular hypertension

**DOI:** 10.1371/journal.pone.0187062

**Published:** 2017-10-26

**Authors:** Sonu Kashyap, Gina Warner, Zeng Hu, Feng Gao, Mazen Osman, Yousif Al Saiegh, Karen R. Lien, Karl Nath, Joseph P. Grande

**Affiliations:** 1 Department of Laboratory Medicine & Pathology, Mayo Clinic, Rochester, Minnesota, United States of America; 2 Kogod Aging Center, Department of Anesthesiology, Mayo Clinic, Rochester, Minnesota, United States of America; 3 UT Southwestern Medical School, Dallas, Texas, United States of America; 4 Hannover Medical School, Hannover, Germany; 5 Division of Nephrology & Hypertension, Mayo Clinic, Rochester, Minnesota, United States of America; Max Delbruck Centrum fur Molekulare Medizin Berlin Buch, GERMANY

## Abstract

Renovascular hypertension (RVH) has deleterious effects on both the kidney and the heart. TGF-β signaling through Smad3 directs tissue fibrosis in chronic injury models. In the 2-kidney 1-clip (2K1C) model of RVH, employing mice on the 129 genetic background, Smad3 deficiency (KO) protects the stenotic kidney (STK) from development of interstitial fibrosis. However, these mice have an increased incidence of sudden cardiac death following 2K1C surgery. The purpose of this study was to characterize the cardiovascular phenotype of these mice. Renal artery stenosis (RAS) was established in Wild-type (WT) and Smad3 KO mice (129 genetic background) by placement of a polytetrafluoroethylene cuff on the right renal artery. Mortality was 25.5% for KO mice with RAS, 4.1% for KO sham mice, 1.2% for WT with RAS, and 1.8% for WT sham mice. Myocardial tissue of mice studied at 3 days following surgery showed extensive myocyte necrosis in KO but not WT mice. Myocyte necrosis was associated with a rapid induction of *Ccl2* expression, macrophage influx, and increased MMP-9 activity. At later time points, both KO and WT mice developed myocardial fibrosis. No aortic aneurysms or dissections were observed at any time point. Smad3 KO mice were backcrossed to the C57BL/6J strain and subjected to RAS. Sudden death was observed at 10–14 days following surgery in 62.5% of mice; necropsy revealed aortic dissections as the cause of death. As observed in the 129 mice, the STK of Smad3 KO mice on the C57BL/6J background did not develop significant chronic renal damage. We conclude that the cardiovascular manifestations of Smad3 deficient mice are strain-specific, with myocyte necrosis in 129 mice and aortic rupture in C57BL/6J mice. Future studies will define mechanisms underlying this strain-specific effect on the cardiovascular system.

## Introduction

Atherosclerotic renal artery stenosis is an important cause of secondary hypertension. Up to 45% of patients with coronary or aortoiliac disease have RAS [1, 2]. The affected kidney undergoes progressive interstitial fibrosis, tubular atrophy, and interstitial inflammation [3]. The Cardiovascular Outcomes in Renal Atherosclerotic Lesions (CORAL) trial showed that percutaneous transluminal renal angioplasty (TRA) failed to improve renal function, reduce renal or cardiovascular events, or decrease mortality, and 16% of patients progressed to a renal endpoint [4]. Although there has been a renewed emphasis on medical management of patients with atherosclerotic renovascular disease, these patients are at high risk of death, up to 16% annually, largely due to cardiovascular events [5]. New therapeutic approaches for preventing both renal and cardiovascular disease in patients with RVH are clearly needed.

TGF-β signaling through Smad3 plays an important role in both renal and cardiac fibrosis, and therefore may represent a potential therapeutic target for patients with RVH [6, 7]. Smad3 is phosphorylated by cell surface TGF-β receptors and partners with Smad4, leading to the transcription of genes involved in growth regulation, inflammation, and fibrosis [8, 9]. Smad3 has emerged as a critical mediator of TGF-β stimulated extracellular matrix production [10, 11]. Smad3 KO mice have been used to study fibrosis in a number of chronic renal injury models, including ureteric obstruction, diabetic nephropathy, and RAS [12–15]. In vascular injury models, Smad3 limits intimal hyperplasia in response to injury [16]. Heart allografts in Smad3 KO mice develop accelerated intimal hyperplasia with increased influx of adventitial macrophages [17].

In human and experimental RVH, angiotensin II promotes renal and cardiac fibrosis through a TGF-β dependent pathway. In our previous studies using the 2K1C model of RVH, we found that the development of severe renal fibrosis and atrophy in the STK of mice was associated with a significant induction of TGF-β and Smad3 [18].

In Smad3 KO mice on the 129 genetic background, we found that the cuffed kidney was remarkably resistant to the development of interstitial fibrosis, interstitial inflammation, and tubular atrophy, compared to WT mice on the same genetic background [15]. Other investigators have shown that Smad3 KO mice (on the C57BL/6J background) are prone to sudden death due to aortic dissections [19]. During our previous studies, we noted that some of Smad3 KO mice on the 129 genetic background died suddenly, but did not develop aortic aneurysms. The objective of this study was to characterize the cardiovascular phenotype of Smad3 KO mice on the 129 genetic background, with an emphasis on early time points following RAS surgery.

## Materials and methods

### Animal model

129-*Smad3*^*tm1Par*^/J breading pairs were purchased from the Jackson Laboratory (Bar Harbor, ME) and a breeding colony was established and maintained as described previously [15, 20]. Both male and female mice were used and RAS surgery was performed by placement of a polytetrafluoroethylene cuff on the right renal artery of anaesthetized mouse under isoflurane (1.5%). Sham was done without placement of cuff. The animals were given buprenorphine (0.05–0.2 mg/kg) before and then 8–12 hrs post-surgery for pain management. Each animal was given 0.5–1.0ml saline subcutaneously immediately post- surgery. Incision sites were monitored twice daily to insure they remained clean, dry and intact.

We previously determined the effect of Smad3 deficiency on the renal phenotype of mice with 2K1C RVH [15]. In conducting these studies, we noted that Smad3 KO mice with RVH had increased mortality, presumably due to sudden cardiac death mostly with in first two weeks following RAS. The vast majority of the deaths were without antecedent clinical manifestations. To study the cardiovascular events in detail, we included 2 wk and 6 wk mice and did a series of additional studies at early time points, 3 and 7 days following surgery. For survival, we studied 425 mice (153 KO with RAS, 49 KO with sham procedure, 167 WT with RAS, and 56 WT with sham). The animals were closely observed for 24 hrs post-surgery and daily thereafter for any side effects. Criteria for evaluation included decrease in food and/or water intake, decreased activity, muscle rigidity, lethargic movement, infection, neurological problems. However, the vast majority of Smad3 deficient mice in our ongoing studies died suddenly, with no antecedent clinical manifestations. We did identify lethargy and decreased movement in 7 of 153 Smad3 KO animals; these animals were euthanized using CO2 inhalation as per Mayo Clinic IACUC guidelines.

Smad3 KO mice were also backcrossed to the C57BL/6J strain for 10 generations and similar to the 129 background, RAS (N = 8) or sham (N = 4) surgery was performed on WT and KO mice. Animals were harvested at 4 weeks following surgery. All of the animal procedures were performed after the appropriate approval by the Mayo Clinic Institutional Animal Care and Use Committee (IACUC).

### Blood pressure measurement

Blood pressure was measured non-invasively on conscious mice using the volume pressure recording (VPR) sensor technology (CODA 6, Kent Scientific Corporation, Torrington, Connecticut, USA).

### Biochemical analysis

Blood was collected via the inferior vena cava at the time of harvest; plasma was separated by centrifugation and stored at −80°C until the time of assay. Quantitative determination of plasma renin content was done by the radioimmunoassay as described earlier [15, 21].

### Histological and immunohistochemical analysis

The heart tissues were fixed in 10% neutral buffered formalin after excision and weighing and processed using standard techniques. Five μm histological sections were prepared and stained with hematoxylin-eosin (H&E) and Masson’s Trichrome stains. Immunohistochemical staining was performed using antibodies for F4/80 (1:200, Abd serotec Raleigh, NC), collagen III (1:20, SouthernBiotech, Birmingham, AL), alpha SMA (1:500, Abcam, Cambridge, MA), iNOS (1:800, Abcam, Cambridge, MA) and CD206 (1:800, Abcam, Cambridge, MA). All measurements and quantifications were performed in a random blinded fashion using Olympus Bx50 microscope (Olympus Optical Co. Ltd., Buffalo Grove, IL), Micropublisher 3.3 RTV camera (QImaging, Surrey, BC, Canada). The amount of necrosis in each heart section was assessed on H&E sections. Quantitative analysis of % area of fibrosis for trichrome and % positively stained area for F4/80, alpha SMA and collagen III was performed using NIS elements BR 4.13.00 64-bit image analysis system (Nikon Instruments INC., Melville, NY) at 200X magnification. The number of iNOS^+^ cells was counted at 200X and CD206^+^ cells at 400X high field magnification.

### Real-time PCR

Total RNA was extracted from the heart tissues using RNeasy Fibrous Tissue Mini kit (Qiagen, Valencia, CA). RNA quantification was done using spectrophotometry (NanoDrop Technologies, Wilmington, DE). RNA quality was assessed using Agilent 2100 Bioanalyzer (Agilent Technologies, Santa Clara, CA). First-strand cDNA was prepared from total RNA using iScript cDNA synthesis kit (Bio-Rad, Hercules, CA). Real time PCR amplification reactions were performed on a Bio-Rad CFX96 real-time PCR detection system. Commercially available primers were used for *ACTA2* and *GAPDH* (ThermoFisher Scientific, Waltham, MA). The sequences for other primers used are provided in S1 Table.

### Zymography

The protein lysates were prepared using RIPA buffer and quantified using Pierce BCA protein assay kit (Thermo Fisher Scientific). The protein samples were run on the 10% Criterion^TM^ zymogram gel with gelatin (BioRad) and then gel was transferred to the renaturation buffer for 15 minutes with gentle agitation at room temperature. This was repeated four times for a total of one hour. Following this, gel was transferred to the developing buffer overnight at 37°C and then stained with 0.5% Coomassie R-250 in Methanol (40%) and acetic acid (10%). Destaining was done in Methanol (40%) and acetic acid (10%) solution until the desired intensity of the bands was acquired. The acquired image was analyzed for the MMP-2 and MMP-9 band intensity by ImageJ software (http://imagej.nih.gov/ij/).

### Statistical analysis

Data are presented as means ± SEM. t-test or ANOVA performed for comparison between groups and post hoc Tukey/Dunn’s correction was used for multiple comparisons. *P* values < 0.05 were considered as significant. Statistical analyses were performed with GraphPad Prism 6 (GraphPad Software, La Jolla, CA).

## Results

### Mortality of Smad3 KO mice with RVH is higher than that of WT mice

A total of 425 mice on the 129 genetic background were studied for survival (153 KO with RAS, 49 KO with sham procedure, 167 WT with RAS, and 56 WT with sham). Overall mortality was 25.5% for KO mice with RAS, 4.1% for KO sham mice, 1.2% for WT with RAS, and 1.8% for WT sham mice (Table 1). Forty one percent of deaths occurred within 1 week of surgery, 71% within the first 2 weeks, and 87% within the first 3 weeks following surgery.

**Table 1:** Mortality in Smad3 KO and WT mice on the 129 background observed at different time points following RAS or sham surgery.

		Days Post Surgery																				
Strain	Surgery	0	1	2	3	4	5	6	7	9	10	12	13	14	18	19	20	21	24	27	30	42
**KO**	**RAS**	1	1	1	4	1	4	2	2	2	3	2	1	4	1	3	1	1	1	1	2	1
	**Sham**	0	1	0	0	0	0	0	0	0	0	0	0	0	0	0	1	0	0	0	0	0
**WT**	**RAS**	0	0	0	0	0	0	0	0	0	0	0	0	0	1	0	0	0	0	0	0	1
	**Sham**	0	1	0	0	0	0	0	0	0	0	0	0	0	0	0	0	0	0	0	0	0

Since age-matched KO mice weighed 14% less than WT mice [15], we used older KO mice to more closely approximate body weights in the two genotypes. We found that overall mortality was greater in older KO, but not WT mice subjected to RAS—the mean age at surgery of KO mice which died suddenly was 20.9 weeks, whereas the mean age of Smad3 KO mice that survived until the end of the experiment was 16.5 weeks (p<0.0001, S1 Fig).

### Systolic blood pressure was lower in Smad3 KO mice than in WT mice with RVH

**Fig 1:**
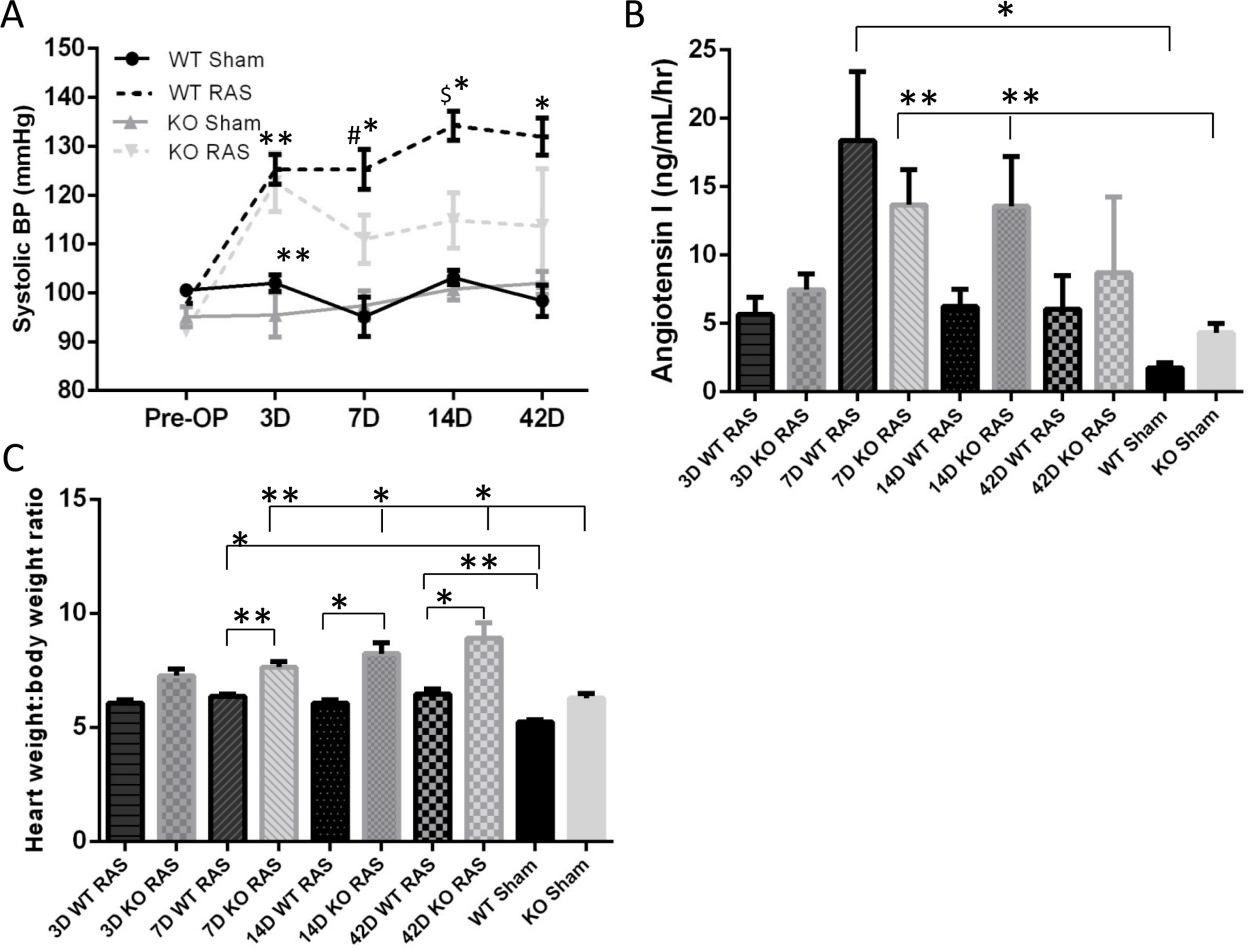
A. Systolic blood pressure in Smad3 KO mice was significantly lower compared to WT mice 7 days after RAS surgery. ^#^p = 0.04, ^$^p = 0.0007 compared to KO RAS mice, *p<0.0001, **p<0.05, compared to respective sham. B. Plasma angiotensin I level in WT and Smad3 KO mice following RAS or sham surgery. C. The heart: body weight ratio is significantly higher in KO RAS mice compared to WT RAS mice at all-time points after day 3. *p<0.0001, **p<0.05.

Systolic blood pressure increased to a similar extent in KO and WT mice at 3 days following RAS surgery. By 7 days, however it was significantly lower in KO than WT mice subjected to RAS, a difference which persisted through 6 weeks of observation (Fig 1A). While plasma angiotensin I production peaked at day 7 in WT RAS mice and declined thereafter, levels in KO RAS mice remained significantly elevated through day 14 (Fig 1B). There was no significant difference in plasma angiotensin I level between WT and KO mice at any time point. Based on these data, the difference in plasma angiotensin I level cannot completely account for the difference in mortality between WT and KO mice. Despite lower blood pressure in KO RAS mice, the heart weight to body weight ratio was higher in KO mice than WT mice with RVH at all time points after day 7 (Fig 1C).

### Histologic findings in hearts of Smad3 KO mice with RVH

**Fig 2:**
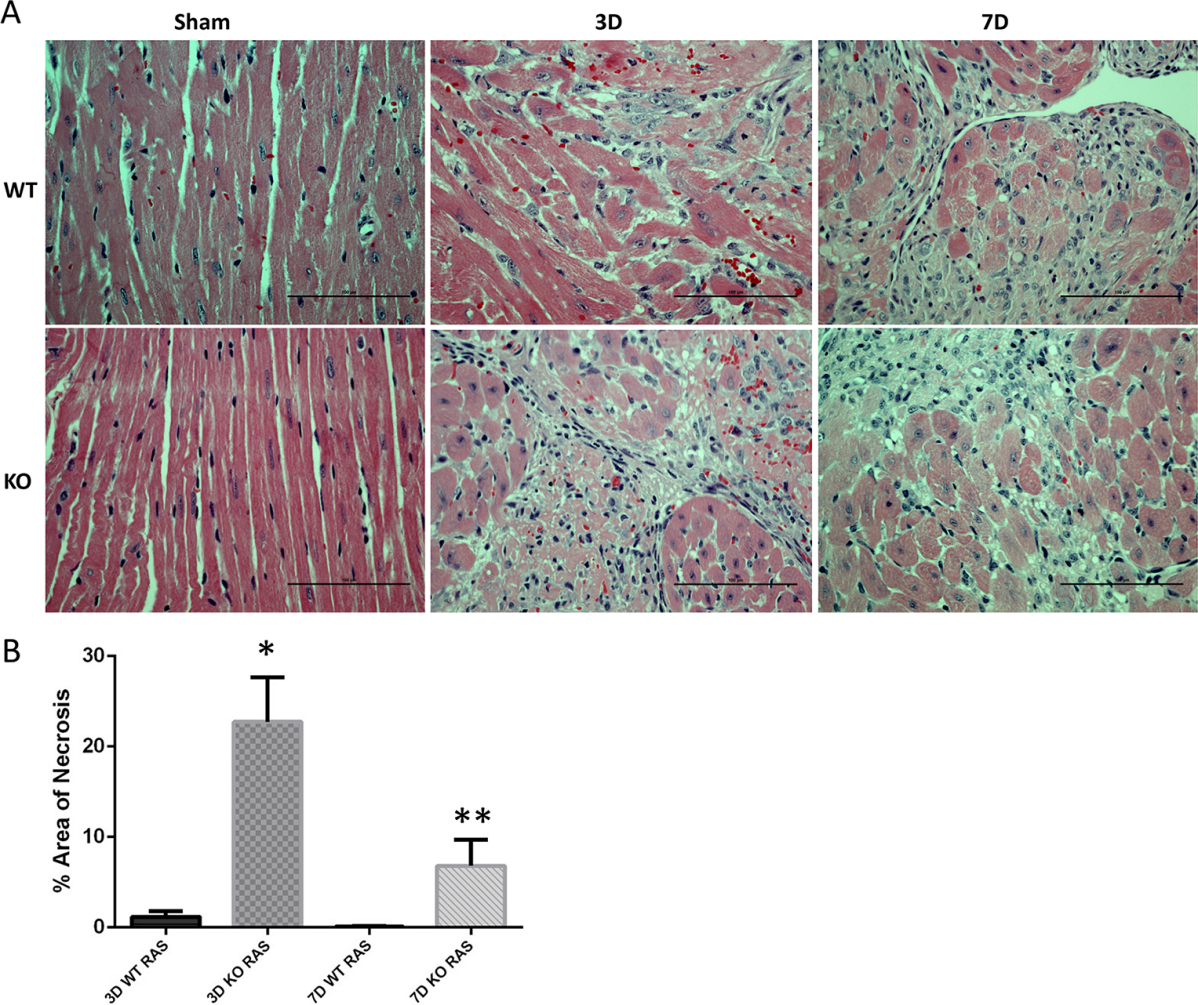
A. Representative histological images showing extensive myocyte necrosis in Smad3 KO RAS mice (stained with H&E, 400X magnifications). Scale bar represents 100μm. B. Myocyte necrosis, as assessed by the % of total myocardial surface area with necrosis in WT and KO RAS mice. *p = 0.0001 **p = 0.02, compared to respective WT time point.

Hearts isolated from KO mice at 3 days following RAS surgery showed extensive myocyte necrosis (Fig 2A), involving over 20% of the cross sectional area of myocardium (22.7±4.9%, Fig 2B). At the same time point, WT RAS hearts showed mild and patchy myocyte necrosis (Fig 2A), involving less than 2% of the cross sectional area of the myocardium (1.2±0.6%, Fig 2B). At 7 days following surgery, myocyte necrosis was focal in KO RAS hearts involving 7% of the myocardial surface area and minimal in WT RAS hearts (Fig 2B).

**Fig 3:**
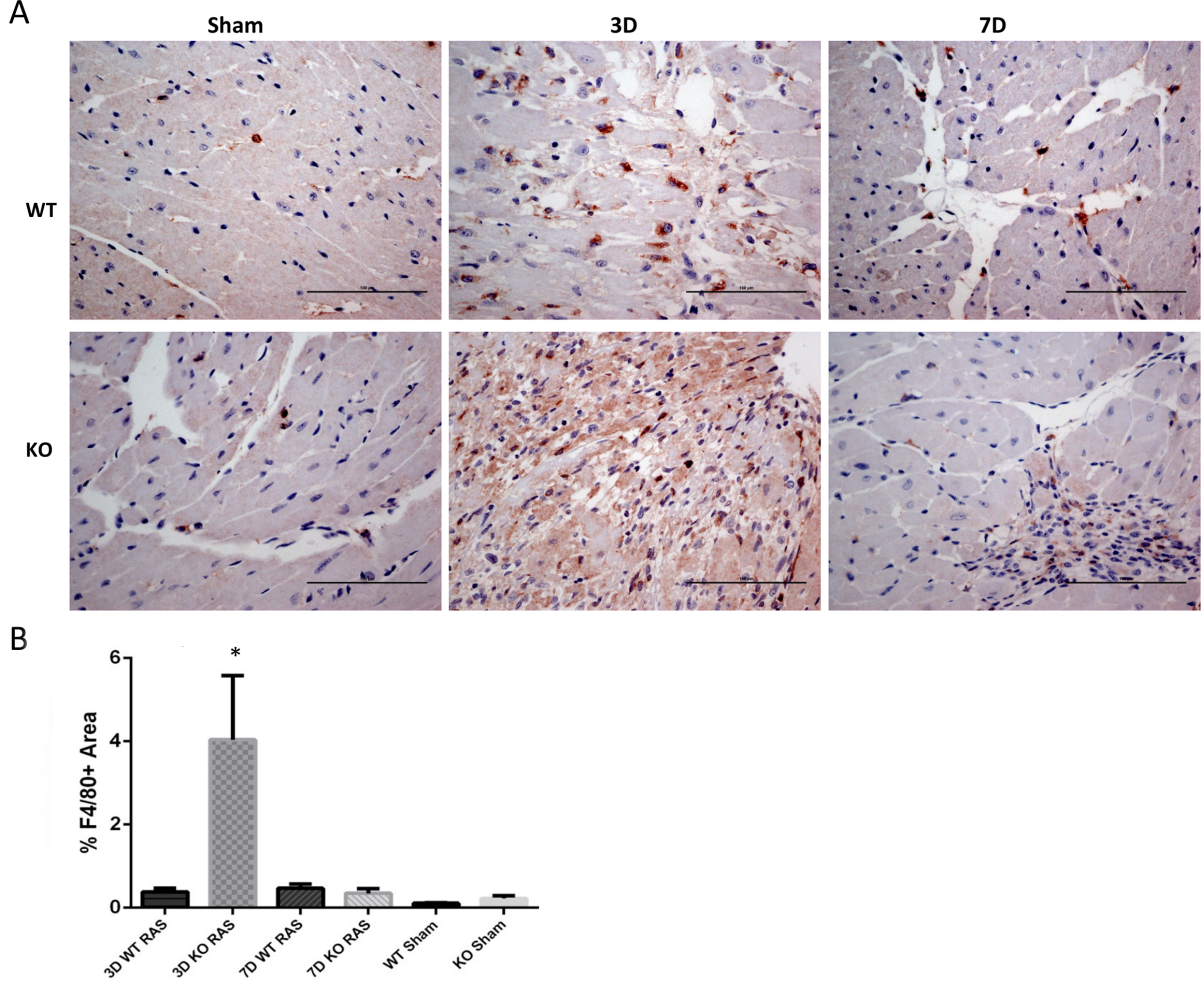
A. Representative histological images showing the anti-F4/80 staining at day 3 and 7 following RAS surgery and sham (200X magnification). Scale bar represents 100μm. B. Graph showing the % area stained positive for F4/80 in WT and KO mice following surgery. *p ≤0.001 compared to respective WT RAS, KO sham and 7D KO RAS mice.

**Fig 4:**
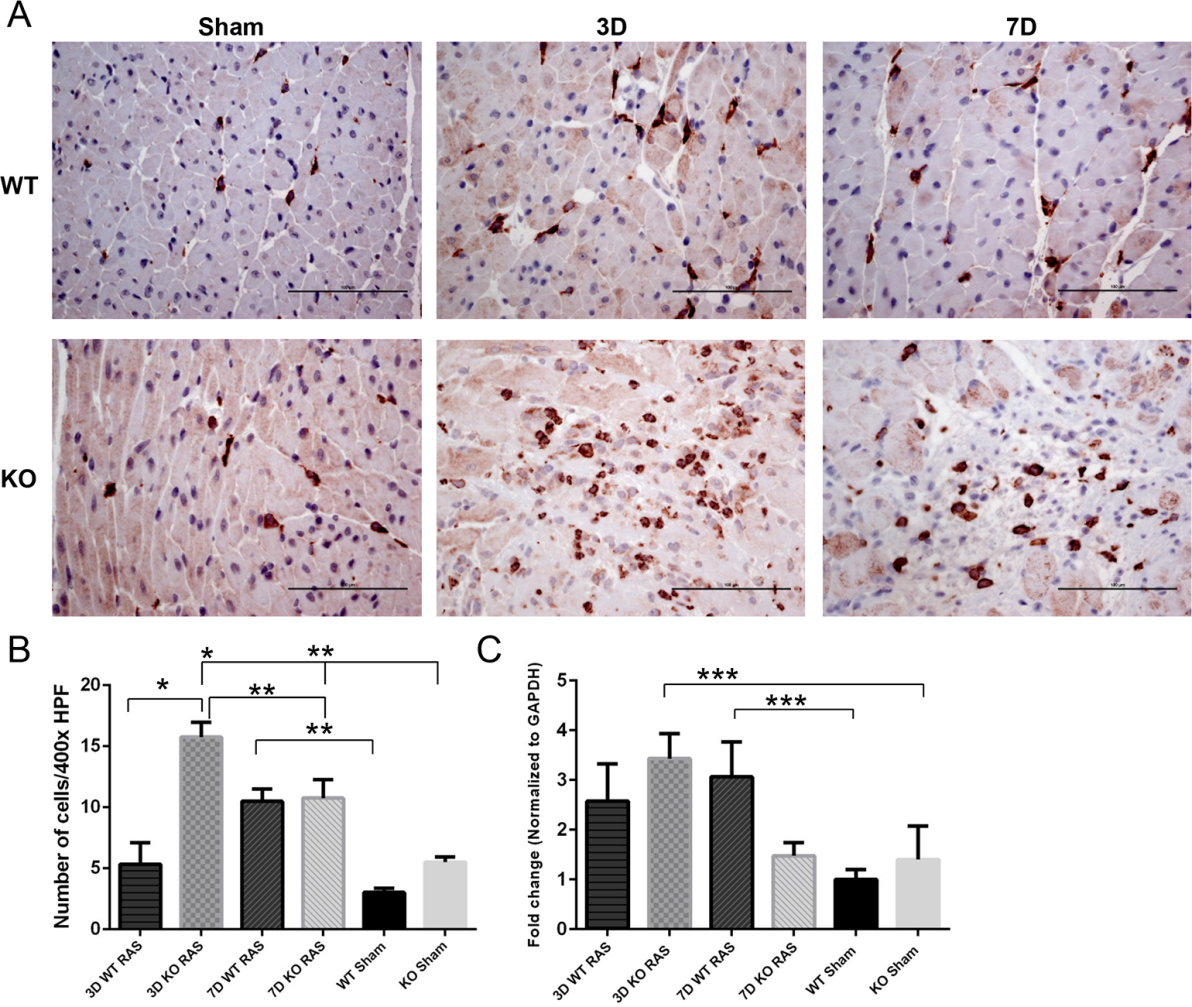
A. Representative histological images showing the anti-CD206 staining at day 3 and 7 following RAS surgery and sham (400X magnification). Scale bar represents 100μm. B. Graph showing the number of cells positive for CD206 / 400x high power field in WT and KO mice following surgery. C. *Cd206* mRNA expression at day 3 and 7 following surgery. *p <0.0001, **p<0.01, ***p<0.05.

**Fig 5:**
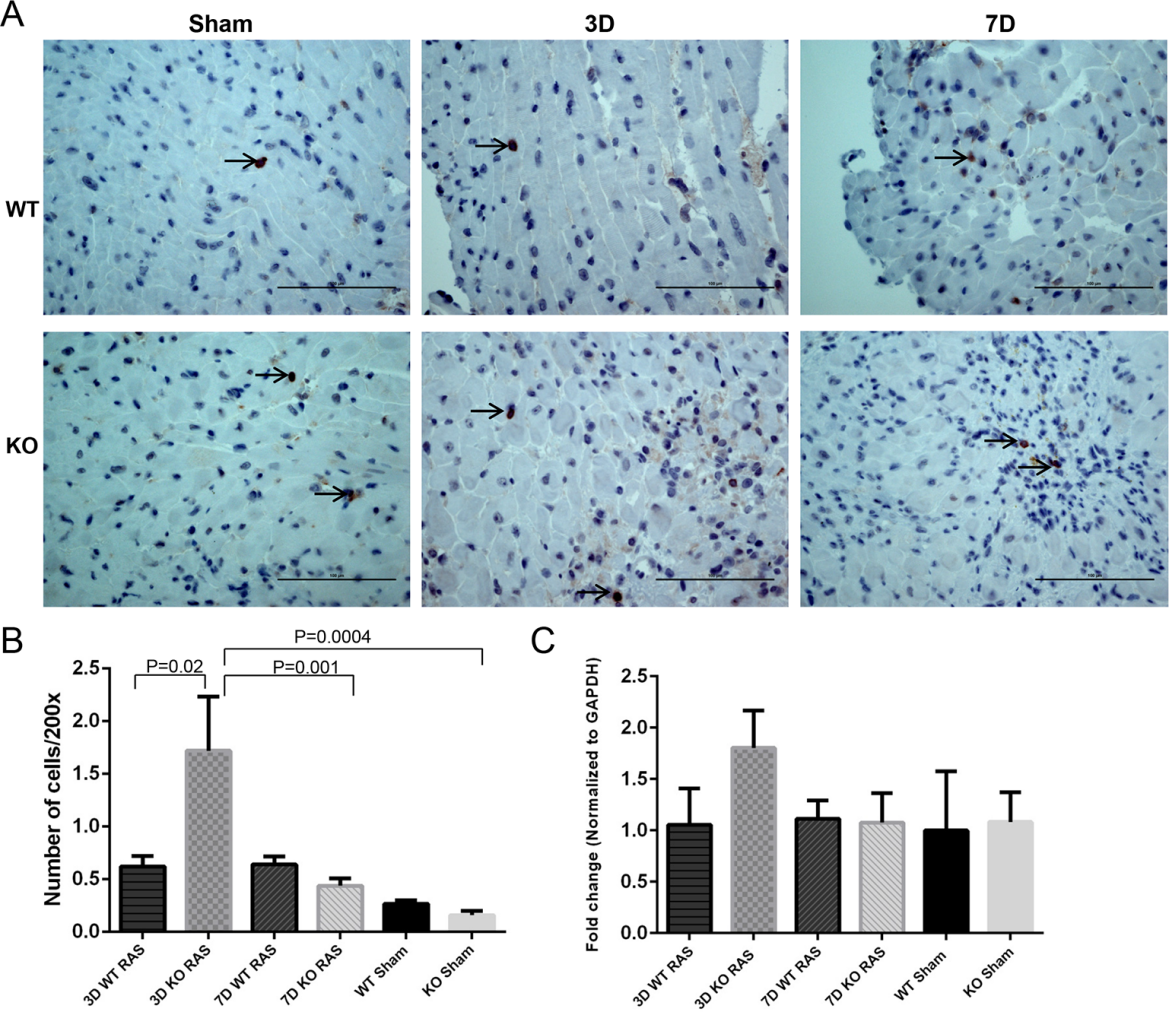
A. Representative histological images showing the anti-iNOS staining at day 3 and 7 following RAS surgery and sham (400x magnification). The positive cells shown with arrow heads. Scale bar represents 100μm. B. Graph showing the number of cells positive for iNOS / 200X in WT and KO mice following surgery. C. *iNos* mRNA expression at day 3 and 7 following surgery.

The myocyte necrosis observed at 3 days following RAS surgery was associated with a significant increase in influx of macrophages as assessed by % surface area positively stained for F4/80 in KO RAS hearts (Fig 3A). The % surface area positive for F4/80 declined to near baseline levels by 1 week following surgery (Fig 3B). The number of CD206^+^ cells was significantly increased in KO RAS hearts, compared to WT RAS hearts at 3 days following surgery (Fig 4). At later time points, the number of CD206^+^ cells was similar in WT and KO mice. However, mRNA expression of *Cd206* showed a significant increase in KO RAS mice compared to KO sham at day 3, but we did not observe significant difference between WT and KO RAS (Fig 4C). The number of infiltrating iNOS^+^ cells was relatively low at all the time points (Fig 5). We did not see any difference in *iN*os expression in either group (Fig 5C).

### *Ccl2 e*xpression is transiently increased in Smad3 KO mice with RVH

**Fig 6:**
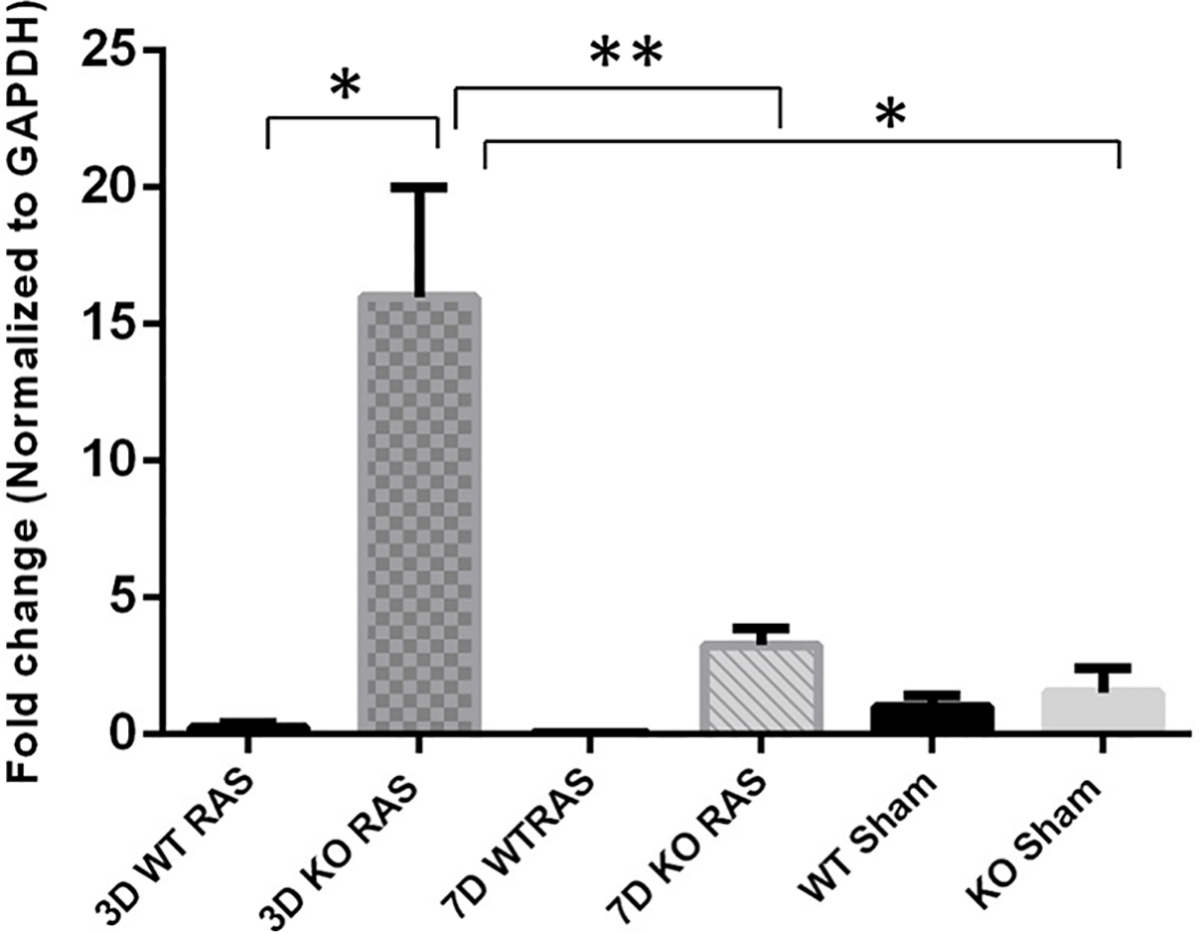
Smad3 KO RAS mice showed significant induction in *Ccl2* mRNA expression at day 3 following RAS. *p<0.0001, **p = 0.0002.

The influx of inflammatory cells was associated with a significant induction of *Ccl2* mRNA expression in KO myocardium 3 days following RAS surgery, as compared to WT RAS heart tissue (Fig 6). Similarly, in parallel with the F4/80 data, *Ccl2* mRNA expression decreased significantly in KO hearts one week following RAS.

### Myocardial fibrosis is similar in Smad3 KO and WT RAS mice

**Fig 7:**
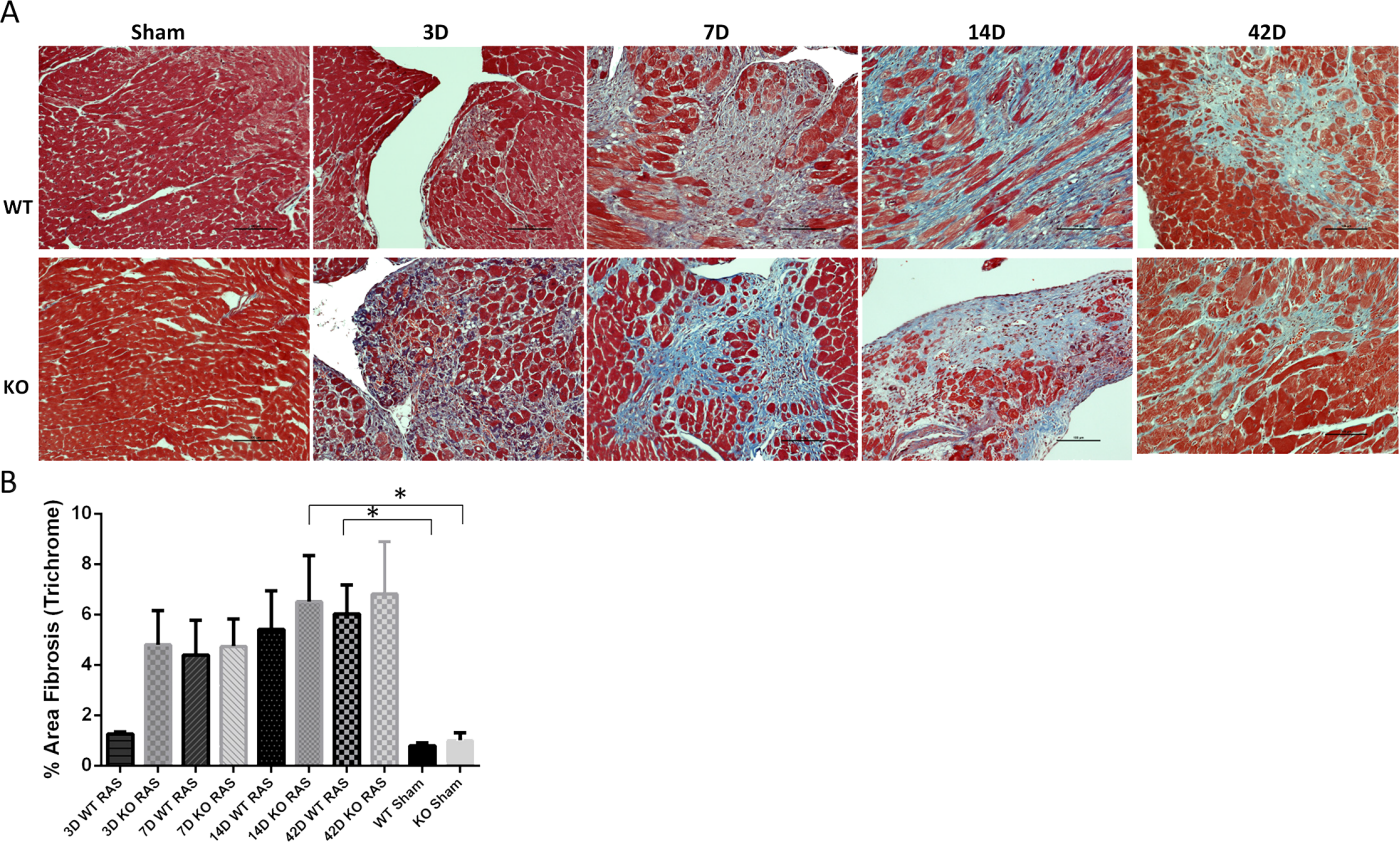
A. Representative histological images showing the myocardial remodeling as assessed by trichrome stain (200X magnification) in WT and KO mice following RAS and sham surgery. Scale bar represents 100μm. B. Graph showing % area fibrosis as assessed by trichrome positive staining. *p<0.01.

Myocardial remodeling, characterized by deposition of trichrome positive extracellular matrix was evident in both KO (4.8±1.4% surface area) and WT (1.26±0.1%, Fig 7B) mice at day 3 following RAS. At later time points, the extent of myocardial remodeling was similar in KO and WT RAS hearts (Fig 7A and 7B).

**Fig 8:**
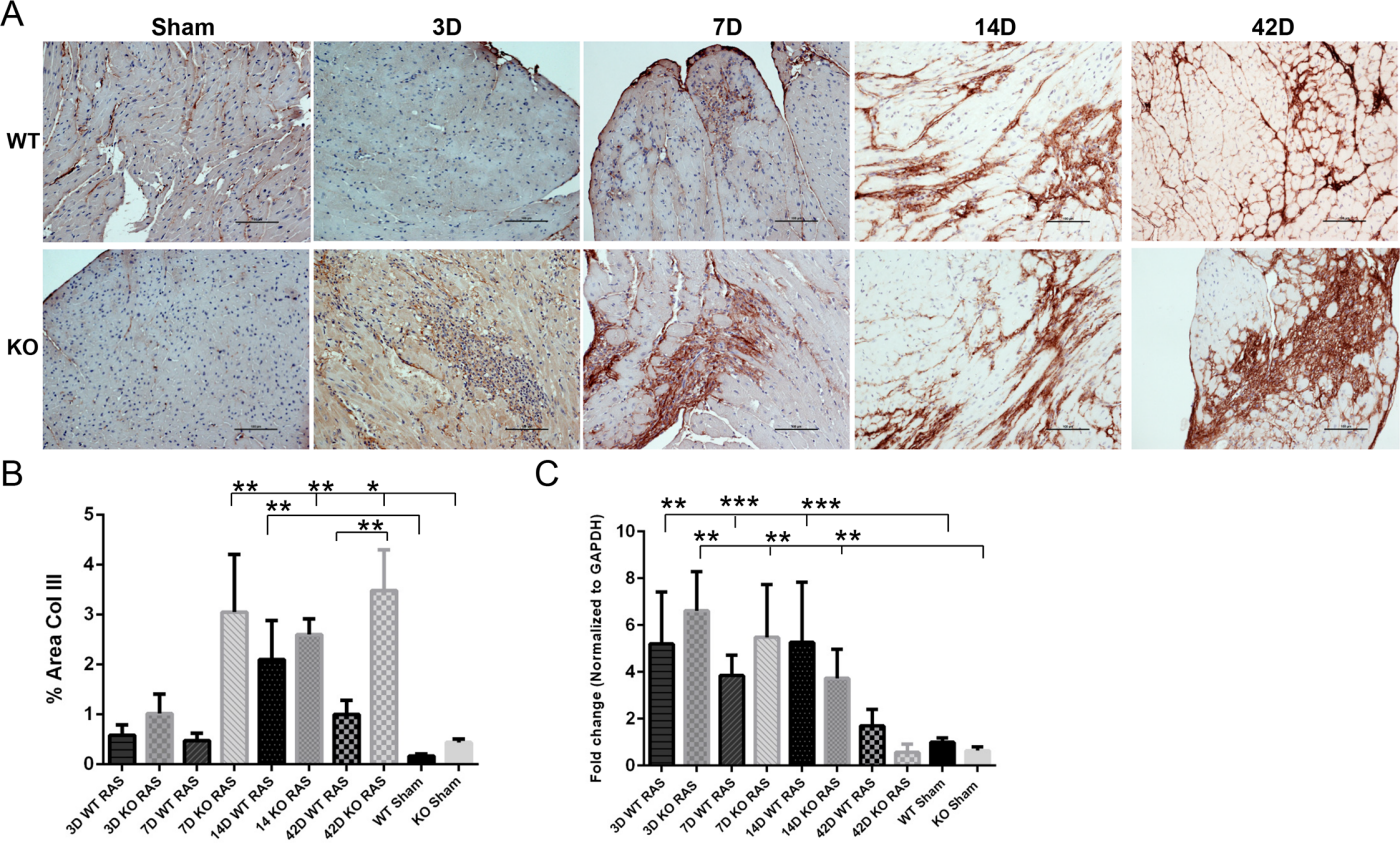
A. Representative histological images showing anti-collagen III staining (200X magnification) in WT and KO mice following RAS and sham surgery. Scale bar represents 100μm. B. Graph showing % area positive for collagen III staining. C. *Col3A1* mRNA expression at day 3, 7, 14 and 42 following surgery. *p<0.0001, **p<0.01, ***p<0.05.

At 7 days following RAS surgery, significant collagen III deposition (as assessed by quantitative analysis of Collagen III immune-histochemical stains) was observed Smad3 KO but not WT myocardium (Fig 8B). By 14 days post-surgery, collagen III deposition was increased to similar extent in myocardium of both WT and Smad3 KO RAS mice (Fig 8A and 8B). At 6 weeks following surgery, however, collagen III deposition returned to near baseline levels in WT mice but remained significantly elevated in KO RAS mice (Fig 8B). Both WT and KO RAS showed a significant increase in *Col3A1* mRNA expression at day 3, 7 and 14 compared to their respective sham.

**Fig 9:**
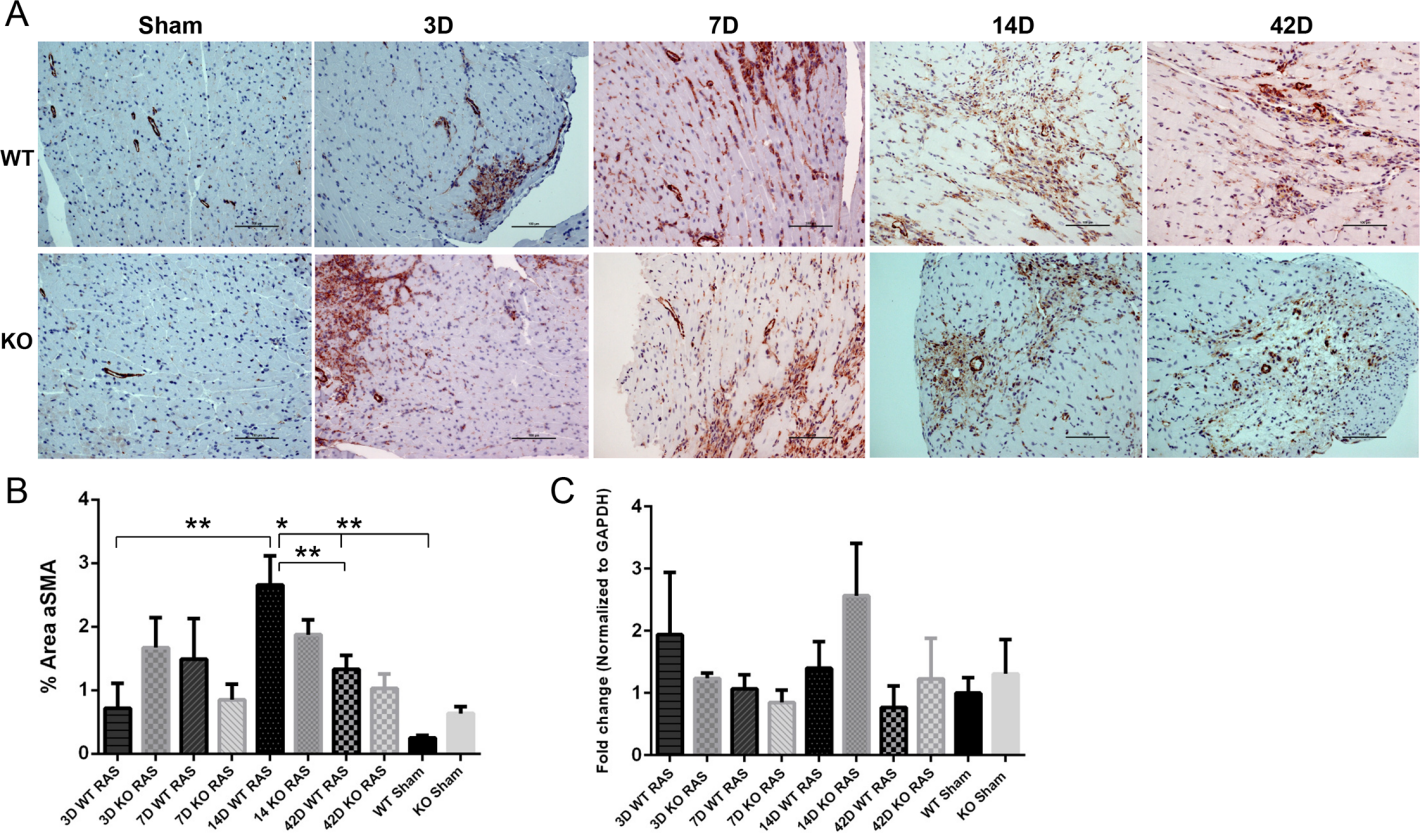
A. Representative histological images showing anti-alpha SMA staining (200X magnification) in WT and KO mice following RAS and sham surgery. Scale bar represents 100μm. B. Graph showing % area positive for alpha SMA staining. C. *Acta2* mRNA expression at day 3, 7, 14 and 42 following surgery. *p<0.0001, **p<0.05.

Development of myocardial fibrosis was associated with increased alpha SMA expression by histology in both WT and KO mice with RVH (Fig 9A), though there was no significant difference between KO RAS and KO sham mice at any time point (Fig 9B). We did not find any change in αSMA *(Acta2)* mRNA expression in any group (Fig 9C).

### MMP9 activity is increased in Smad3 KO RAS hearts

**Fig 10:**
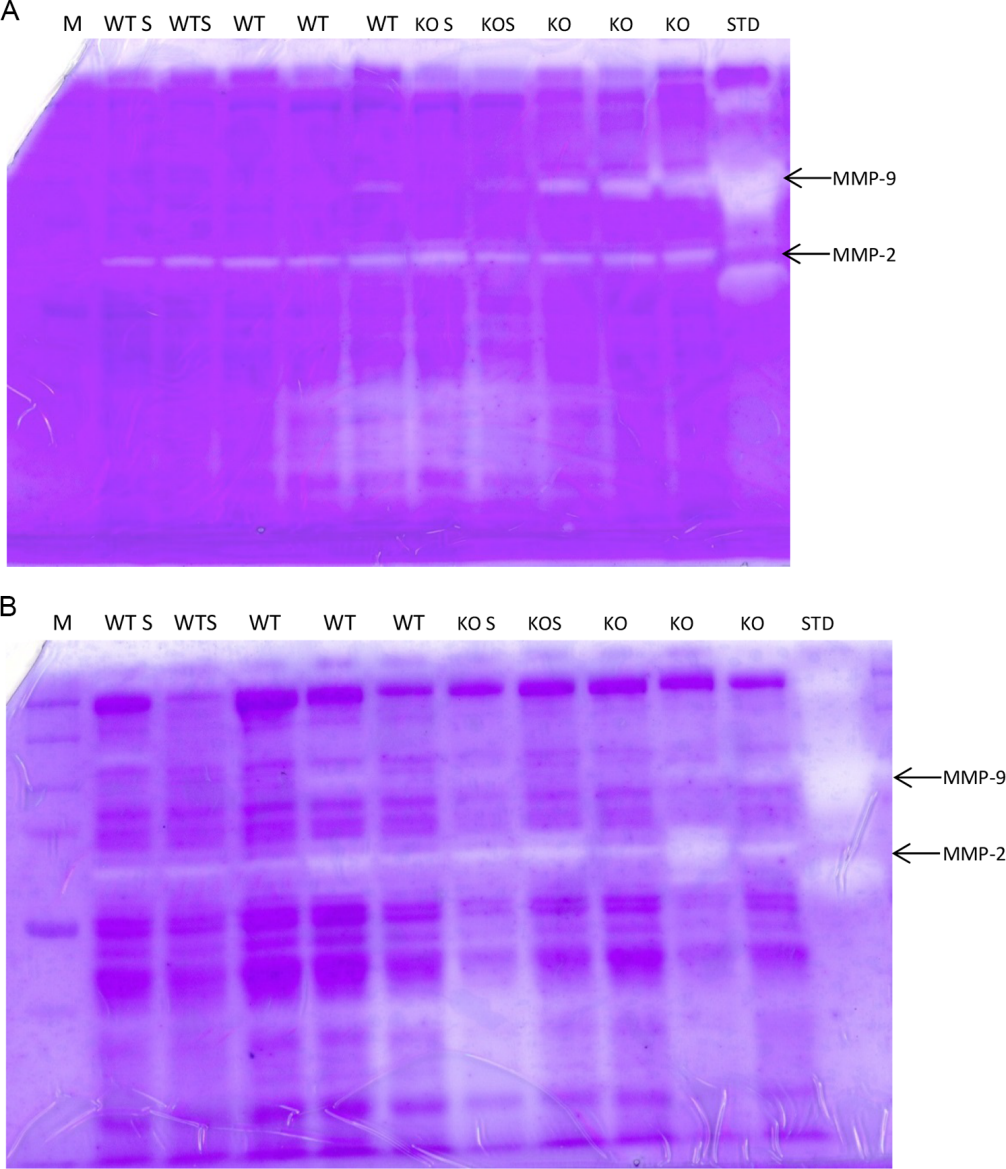
A. MMP activity, as assessed by gel zymography in WT and KO at 3 days following surgery. Higher MMP-9 activity found in KO RAS mice. B. MMP activity at 7 days following surgery. M = marker, KO S = KO sham, WT S = WT sham, KO = KO RAS, WT = WT RAS, STD = MMP-9 and MMP-2 standard.

MMP-9 activity, as assessed by gel zymography, was increased in myocardial tissue isolated from KO RAS mice, but not in WT RAS mice at 3 days following surgery (Fig 10A). MMP-9 activity returned to baseline levels after one week in KO RAS mice (Fig 10B). There was no difference observed in MMP-2 activity.

### Smad3 KO mice on C57BL/6J background developed aortic aneurysms

**Fig 11:**
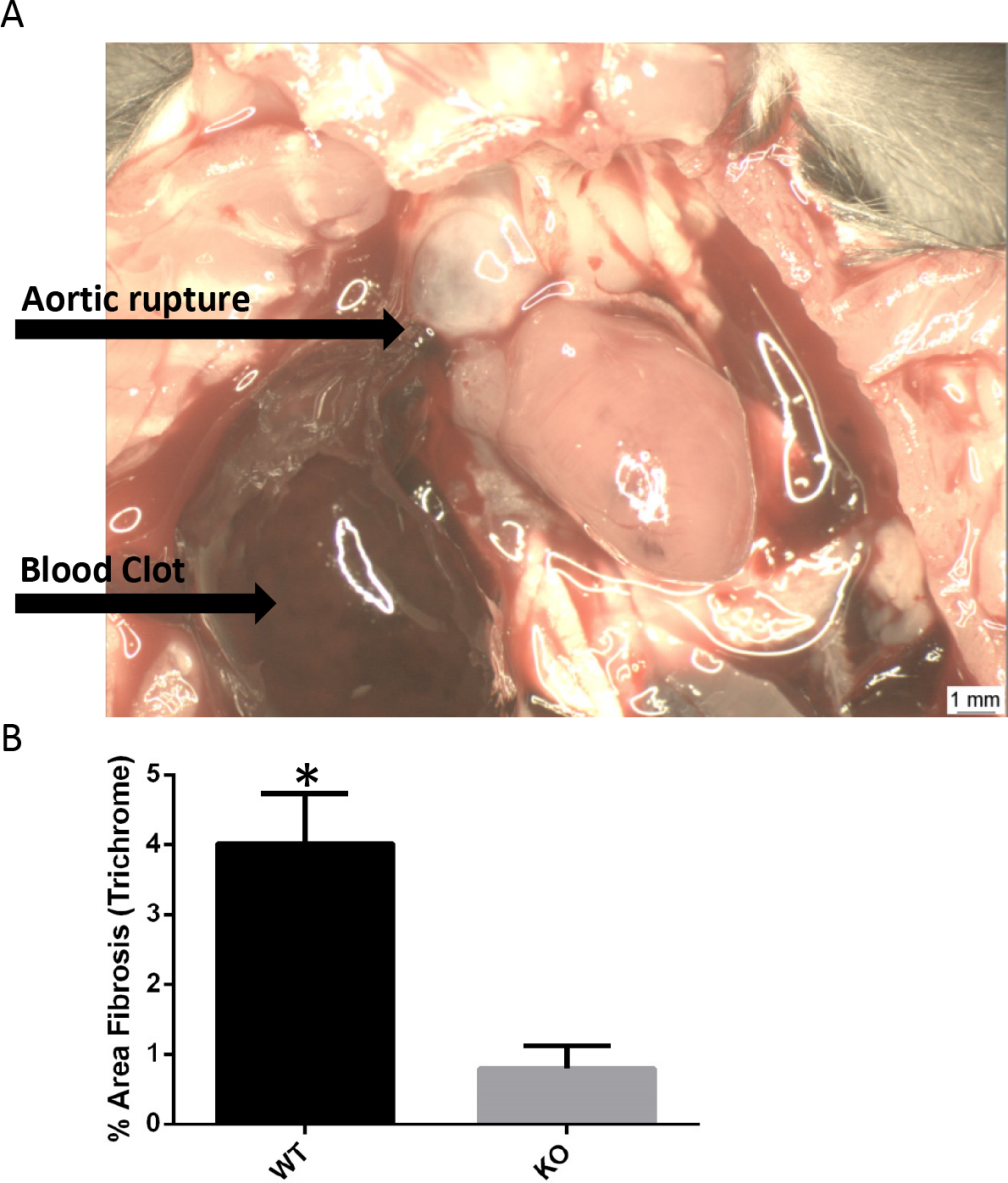
A. Photomicrograph showing the blood clot in the thoracic cavity and aortic rupture in Smad3 KO mice on C57BL/6J background. B. Smad3 KO RAS heart on C57BL/6J background showed significantly less fibrosis compared to WT RAS heart. *p = 0.009.

Similar to the Smad3 KO mice on the 129 background, mortality was observed in KO RAS mice on C57BL/6J background (5 out of 8 62.5%) due to sudden death at 10–14 days following surgery. Aortic ruptures were observed in the mice that were available for necropsy (N = 2, Fig 11A). Out of 4 sham KO mice, 1 mouse developed an aortic aneurysm (without dissection). The KO RAS mice showed significantly less cardiac fibrosis compared to WT RAS mice (Fig 11B). As we previously observed in RAS mice on the 129 background, the STK of C57BL/6J mice did not develop significant chronic renal damage (3.75±2.9% atrophy) compared to WT (73.3±15.4% atrophy).

## Discussion

These studies provide insight into the unexpected cardiac mortality in Smad3 KO mice subjected to RVH. Our previous study focused on the protective effect of Smad3 deficiency on the STK of mice with RAS at 2 and 6 weeks following surgery; the heart was not analyzed in a systematic fashion [15]. In the current survival study, we report that 39 deaths occurred in 153 Smad3 KO mice with RAS (25.5%) whereas 2 deaths were found in 167 WT mice with RAS. Most of the deaths in Smad3 KO mice subjected to RAS occurred within the first 2 weeks following surgery and were associated with extensive myocyte necrosis. Myocyte necrosis was associated with rapid induction of *Ccl2*, influx of macrophages and induction of MMP9 activity. WT RAS mice showed only isolated foci of myocyte necrosis at 3 days following surgery. At this time point, there were no significant differences in blood pressure or angiotensin I production between Smad3 KO and WT RAS mice, indicating that myocyte necrosis in Smad3 KO mice cannot be attributed to hypertension alone or to increased systemic angiotensin I levels. Smad3 KO mice infused with angiotensin II become hypertensive, but develop less cardiac hypertrophy, inflammation, and left ventricular dysfunction than WT mice [22]. However, Smad3 KO mice subjected to transverse aortic constriction develop cardiac hypertrophy, although there is less myocardial fibrosis [23]. Mice with an inducible dominant negative mutation in TBRII and subjected to transverse aortic constriction show less collagen deposition but develop LV dilation and dysfunction [24]. TGF-β has a protective effect on the myocardium following ischemia-reperfusion injury, at least in part through downregulation of matrix metalloproteinase activity in a Smad3 dependent fashion [25, 26].

We did not observe any significant aortic pathology in Smad3 KO RAS mice on the 129 background. However, mutations in SMAD3 have been identified in up to 2% of patients with familial thoracic aneurysms leading to acute aortic dissection [27]. Patients with the Loeys-Dietz syndrome have mutations in receptors for TGF-β (TGFβR1 and TGFβR2) and are prone to develop aortic dissections [28]. Angiotensin II infusion into Smad3 KO mice on the C57BL/6J background has been used as a model for development of aortic aneurysms and aortic dissections [29–32]. These studies indicate that development of aneurysms is due to Ang II-mediated macrophage infiltration and upregulation of NOS2 (inducible nitric oxide synthase), matrix metalloproteinases (MMP) 2 and 9 rather than hypertension alone [29, 33]. Along these lines, targeted disruption of the MMP9 gene in bone marrow derived cells protects mice fed a high fat diet and infused with angiotensin II from development of aortic aneurysms [34].

When we backcrossed the Smad3 KO mice to the C57BL/6J background, we were able to confirm that Smad3 KO C57BL/6J RAS mice develop aortic dissections, as previously reported by others. The myocardium of C57BL/6J RAS mice did not develop significant necrosis, although we did not extensively study early time points following surgery. This observation is in accord with other investigators who have shown that the C57BL/6J strain is more resistant to myocardial remodeling following angiotensin II infusion than C57BL/6N mice, an effect that may be related to reduced M2 polarization of macrophages in C57BL/6 mice [35]. Similar to our observations in 129 mice, the stenotic kidney of Smad3 KO C57BL/6J mice was protected from development of atrophy and fibrosis, compared to WT RAS mice. We propose that macrophage mediated inflammation and MMP activity may be responsible for the aortic phenotype observed in Smad3 KO mice on the C57BL/6J background and for the cardiac phenotype observed in Smad3 KO mice on the 129 background. Future studies will define a role for early macrophage influx and MMP activity in the development of myocardial necrosis in Smad3 KO RAS mice on the 129 background, akin to the reported role for macrophage mediated influx on the development of aortic aneurysms in Smad3 KO mice on the C57BL/6J background.

Although we believe that this is the first study to document cardiac lesions in Smad3 KO mice subjected to RVH, there are several limitations. First, although we performed the present study with main focus on the early cardiac events following RAS, the survival study in part was a retrospective study which was not designed to identify cardiac pathology lesions. For this reason, we were not able to evaluate some of the hearts from mice that died suddenly. Second, additional studies are needed to define a mechanistic role for macrophage influx and MMP-9 activity in the development of myocyte necrosis in Smad3 KO RAS mice.

We conclude that, despite a remarkable protective effect on the kidney, Smad3 KO mice with RVH develop myocardial necrosis and, in those that survive, cardiac fibrosis. Our initial studies do not demonstrate any qualitative or quantitative differences between Smad3 KO and WT mice with respect to cardiac fibrosis which develops at later time points. Our preliminary studies on Smad3 KO mice on C57BL/6J background confirmed the presence of aortic dissection as reported by others. Based on these considerations, we conclude that the cardiovascular manifestations of Smad3 deficiency in mice with RVH are strain specific.

## Supporting information

S1 Fig Showing the significantly higher age observed in dead mice compared to survived Smad3 KO mice. (PDF)

S1 Table Sequences of primers used in the study. (PDF)
