# Supplementary material for: Cardiovascular phenotype in Smad3 deficient mice with renovascular hypertension
Source: PLoS One. 2017 Oct 26;12(10):e0187062. doi: 10.1371/journal.pone.0187062 (PMC5658153; doi:10.1371/journal.pone.0187062)

S1 Fig. Showing the significantly higher age observed in dead mice compared to survived Smad3 KO mice

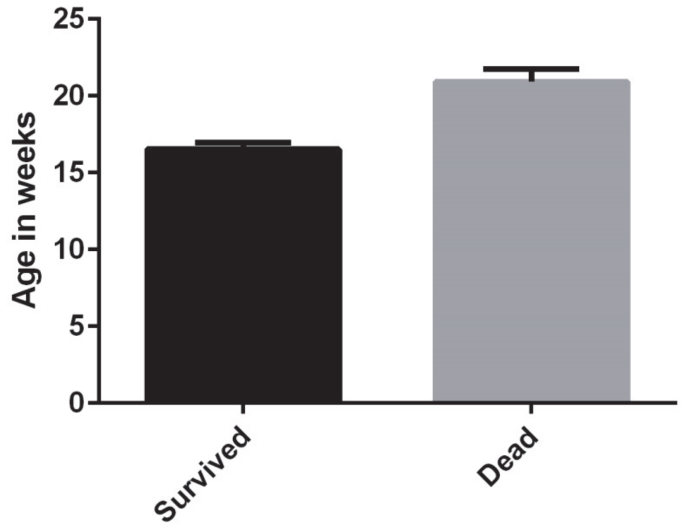

Supplement: S1 Fig — (PDF) [file pone.0187062.s001.pdf]
