# Supplementary material for: Cardiovascular phenotype in Smad3 deficient mice with renovascular hypertension
Source: PLoS One. 2017 Oct 26;12(10):e0187062. doi: 10.1371/journal.pone.0187062 (PMC5658153; doi:10.1371/journal.pone.0187062)
Supplement: S1 Table — (PDF) [file pone.0187062.s002.pdf]

S1 Table. Sequence of primers used in the study

Ccl2
Forward: 5’-TGGATGCTCCAGCCGGCAACT-3’
Reverse: 5’-AGCACCAGCACCAGCCAACTC-3’
Cd206
Forward: 5’- CCA GCT CGG ATA TGA GCC AA -3’
Reverse: 5’- CTG GGG TTC CAT CAC TCC AC -3’
iNos
Forward: 5’- TGG CTC GCT TTG CCA CGG AC -3’
Reverse: 5’- GCT GCG ACA GCA GGA AGG CA -3’
Col3A1
Forward- 5’ –GAG GGG CTC CTG GTG AGC GA-3’
Reverse-5’-ATG TCC CTG CGG TCC AGG CT -3’
